# Erratum to: Clinical metagenomic identification of *Balamuthia mandrillaris* encephalitis and assembly of the draft genome: the continuing case for reference genome sequencing

**DOI:** 10.1186/s13073-015-0257-9

**Published:** 2016-01-11

**Authors:** Alexander L. Greninger, Kevin Messacar, Thelma Dunnebacke, Samia N. Naccache, Scot Federman, Jerome Bouquet, David Mirsky, Yosuke Nomura, Shigeo Yagi, Carol Glaser, Michael Vollmer, Craig A. Press, Bette K. Kleinschmidt-DeMasters, Samuel R. Dominguez, Charles Y. Chiu

**Affiliations:** Department of Laboratory Medicine, University of California, 185 Berry Street, San Francisco, 94107 CA USA; UCSF-Abbott Viral Diagnostics and Discovery Center, San Francisco, 91407 CA USA; Children’s Hospital Colorado and University of Colorado School of Medicine, Aurora, CO USA; California Department of Public Health, Richmond, CA USA; Kaiser Permanente Hospital, Oakland, CA USA; John Muir Hospital, Walnut Creek, CA USA; Department of Medicine, Division of Infectious Diseases, University of California, San Francisco, 94107 CA USA

## Erratum

In the original article [1], there was an error in one of the author names. The author name Bette K. Kleinschmidt-DeMasters was incorrectly spelt as Bette K. Klenschmidt-DeMasters. This has now been corrected in the published article and the publisher apologises for any inconvenience caused.
